# Nessi: An EEG-Controlled Web Browser for Severely Paralyzed Patients

**DOI:** 10.1155/2007/71863

**Published:** 2007-09-10

**Authors:** Michael Bensch, Ahmed A. Karim, Jürgen Mellinger, Thilo Hinterberger, Michael Tangermann, Martin Bogdan, Wolfgang Rosenstiel, Niels Birbaumer

**Affiliations:** ^1^Department of Computer Engineering, University of Tübingen, 72076 Tübingen, Germany; ^2^Institute of Medical Psychology and Behavioral Neurobiology, University of Tübingen, Gartenstrasse 29, 72074 Tübingen, Germany; ^3^Graduate School of Neural and Behavioral Sciences, International Max Planck Research School, University of Tübingen, 72076 Tübingen, Germany; ^4^Fraunhofer FIRST, Intelligent Data Analysis Group, 12489 Berlin, Germany; ^5^Department of Computer Engineering, University of Leipzig, 04103 Leipzig, Germany; ^6^Human Cortical Physiology Unit, National Institute of Neurological Disorders and Stroke (NINDS), National Institutes of Health (NIH), Bethesda, MD 20892, USA

## Abstract

We have previously demonstrated that an EEG-controlled web browser based on self-regulation of slow cortical potentials (SCPs) enables severely paralyzed patients to browse the internet independently of any voluntary muscle control. However, this system had several shortcomings, among them that patients could only browse within a limited number of web pages and had to select links from an alphabetical list, causing problems if the link names were identical or if they were unknown to the user (as in graphical links). Here we describe a new EEG-controlled web browser, called Nessi, which overcomes these shortcomings. In Nessi, the open source browser, Mozilla, was extended by graphical in-place markers, whereby different brain responses correspond to different frame colors placed around selectable items, enabling the user to select any link on a web page. Besides links, other interactive elements are accessible to the user, such as e-mail and virtual keyboards, opening up a wide range of hypertext-based applications.

## 1. INTRODUCTION

**Figure 1:**
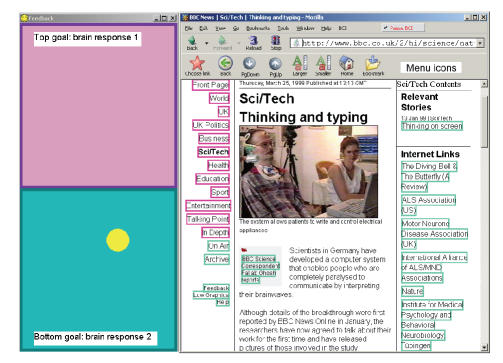
Web surfing with Nessi. Colored in-place link markers correspond to brain responses of the user, which are shown as goals in the BCI feedback window (left). After a page has loaded, the link markers are applied to a set of menu icons (top), allowing the user to choose a link, go back, scroll down the page, and use other configurable options. The number of goals and accordingly link marker colors can be increased for multiclass BCIs.

Neurological diseases such as amyotrophic lateral sclerosis (ALS), Guillain-Barré syndrome, or brainstem stroke can lead to severe or total motor paralysis often referred to as “locked-in syndrome”, where the intact intellect is locked into a paralyzed body. One of the most terrifying aspects of this “locked-in syndrome” is that the loss of muscle control prevents the expression of even the most basic needs. Conventional augmentative communication devices, which depend on some rudimental muscle control, may not be feasible for patients in the end stage of ALS if they have no remaining reliable muscle control. Thus, the final option for restoring communication to those patients is to provide the brain with a nonmuscular communication and control channel, a direct brain-computer interface (BCI) for conveying messages and commands to the external world. In the late 1990s, Birbaumer [1, 2] were the first to provide ALS patients with a BCI system, the so-called thought translation device (TTD). The TTD is a noninvasive, EEG-controlled BCI based on regulation of slow cortical potentials (SCPs) which humans can learn to control in an operant conditioning procedure [3]. Further studies have successfully applied sensory-motor EEG rhythms (SMR) [4, 5], P300 evoked potentials [6], and neuronal action potentials [7–9] for different BCI systems. While letter spelling is a state-of-the-art application for BCI systems today, interactive access to the world wide web (WWW) is one of the most promising BCI applications, as it enables severely paralyzed patients to participate in the broad portion of life reflected by the WWW. Karim et al. [10] have recently shown that an EEG-controlled web browser based on self-regulation of SCP can be reliably operated by a locked-in patient suffering from end stage ALS. Moreover, it was shown that this BCI web browser, called Descartes, can help severely paralyzed patients to regain a certain level of autonomy in the interaction with the outside world, and thereby enhance their quality of life [10–12]. However, Descartes had several shortcomings, among them that patients could only browse within a limited number of web pages and were not able to choose a link, if they did not know its link text (as, e.g., in graphical links). Here we introduce a new EEG controlled web browser, called Nessi (neural signal surfing interface), which overcomes these shortcomings. In Nessi, the web browser, Mozilla, was extended by graphical in-place markers, whereby different brain responses correspond to frame colors placed around selectable items, enabling the user to choose any link on a web page (see Figure 1).

## 2. IMPLEMENTATION OF NESSI

The implementation of Nessi within the Mozilla framework allows customization on various levels. The supervisor is able to adjust parameters concerning the user interface, such as the number of frame colors to use (each color representing one class) or the length of the reading pause. Some HTML knowledge is required to create custom virtual keyboards or start pages. With programming experience, dynamic pages (games) or new decision structures can be created. We have included a game (an image consisting of 6 parts has to be “uncovered” by selecting each part separately) to accustom users to the new interface and increase motivation. Nessi is available open source at http://nessi.mozdev.org and contributions are welcome. The following subsections describe three integral aspects of Nessi: (1) graphical display of in-place link markers; (2) construction of finite-state transducers (FSTs) (cf. [13]), which represent internally the brain responses required to select a link; (3) communication with the BCI software that records and processes the EEG signals.

## 2.1. In-place linkmarkers

Colored frames are placed around selectable items on a web page, circumventing any need to maintain a separate presentation of choices (see Figure 1). By default, red frames are selected by producing negative SCP shifts and green frames are selected by the production of positive SCP shifts. As an aid, feedback is displayed at the left rim of the screen by depicting the vertical movement of a cursor that can be moved upwards into a red goal or downwards into a green goal. The user only has to watch the current color of the desired link's frame that indicates the brain response which has to be produced for its selection. By presenting a series of brain responses as indicated by changing the color of the frame around that link, it can be chosen with binary decision neglecting any knowledge about its position in a selection tree.

The advantages of graphical display of in-place link markers on web pages instead of presenting links in an alphabetical list were discussed previously by Mellinger et al. [14] and Karim et al. [12]. In the following, we give a more technical description of the finite state transducers (decision graphs) used to determine the color of the link markers at each selection step.

Nessi's task mode makes it possible for the supervisor to ask the patient which link he/she wishes to choose and to mark that link as a task. This way, the patient's accuracy can be recorded, enabling a comparison of patients' performance between standard spelling tasks and web surfing.

## 2.2. Construction of finite-state transducers

**Figure 2:**
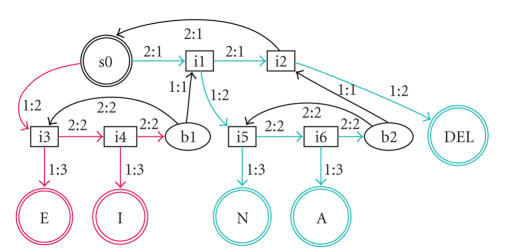
LSP transducer with scanning structure depicting a virtual keyboard with four output letters. The initial state is s0. Transitions are labeled with the classification answer and the logical “depth.” Internal states are marked with an “i” and states allowing for correction of errors by moving back to higher levels (*back* nodes) are marked with a “b.” From the current state, s0, the red states are reached with brain response 1 and the green states are reached with brain response 2.

We represent each step in the selection process as a state transition of an FST [13]. Depending on the FST's input, which is represented by the user's brain response, a transition from one state to the next occurs together with an output. The final states represent the colored marked elements (links on a web page or letters on a virtual keyboard). The input alphabet of the FST is the set of brain responses recognized by the BCI, that is, (1,2) for a typical two-class BCI. However, transducer construction is not limited to two classes and can be adjusted to multiclass BCIs, whereby the number of outgoing transitions of a state matches the number of classes used. The output of the FST represents the depth of the transducer, that is, how close the user is to choosing a final state. This output string can be evaluated by supervisors to see where a patient is having trouble in the selection process. All links pointing to the same URL are merged into one node of the FST. This dramatically reduces the number of choices for many web pages. Link marker colors are determined by finding the input symbol of the first transition representing the shortest path from the current state to the desired link (cf. Figure 2). If the user desires to write the letter “N” on a virtual keyboard, beginning at state s0, the shortest path is 2,1,1 (visiting the states i1 and i5). The first transition thus requires the input symbol “2.” Assigning red markers to brain response 1 and green markers to brain response 2, the “N” is marked green while the current state is s0.

We examined various techniques to construct FSTs for decision making with the objective of quick link or letter selection. In previous studies with ALS patients, the language support program (LSP) [15] and the internet browser Descartes [10] used a “scanning” interface, whereby the brain responses represented *select* or *reject* and the classifier had a strong bias towards *reject* . An example of an LSP transducer is shown in Figure 2.

**Figure 3:**
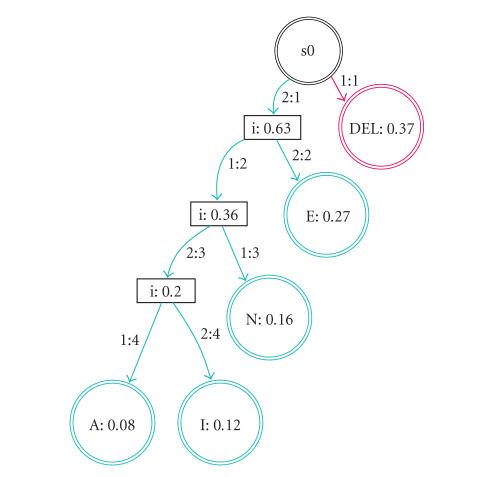
Huffman-coded transducer. The probability that a node will be chosen, based on a language model (or web revisitation patterns for links), is shown inside the node. The *back* nodes have been omitted for clarity. Beginning at state s0, the red state is reached with brain response 1 and the green states are reached with brain response 2.

If the user has reached high accuracy and his/her interaction with the classifier generates two brain responses with equal probability, a Huffman-coded FST [16] might be more efficient. An example is shown in Figure 3. The user is able to correct mistakes by choosing the *back* nodes that are inserted at every second level of the FST.

## 2.3. Communication with BCI software

**Figure 4:**
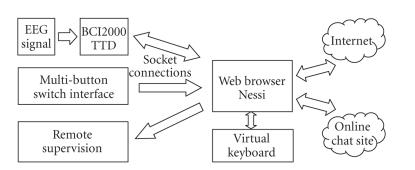
BCI software communicates with Nessi via a socket protocol. Text is entered into web forms or chat sites with a virtual keyboard. Switch interfaces for non-BCI users could be added. Remote supervision can be realized by starting a remote instance of Nessi, which synchronizes with the patient's display.

A communication protocol was defined to interface Nessi with existing BCI systems such as the TTD [2] and BCI2000 [17]. Once a connection is established, the user's favorite bookmark is shown and the links are marked red or green. Feedback of brain responses (e.g., SCP or SMR) can be displayed on the left of the screen in the form of a ball that the user moves into a red or green goal. Each brain response is sent from the BCI to Nessi and used as an input to the FST, which causes a state transition. Once a new web page is selected, Nessi sends a signal to the BCI software to stop feedback and the user has a predefined time (adjusted to the user's reading speed by the supervisor) to read the page. Thereafter, feedback continues and link selection restarts. Other external programs, such as switch interfaces, could also be used to control Nessi. Communication between Nessi and external programs is illustrated in Figure 4.

## 3. E-MAIL

**Figure 5:**
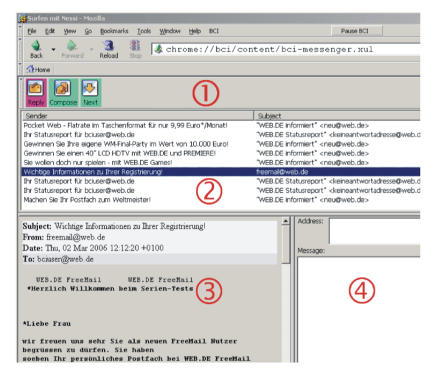
E-mail interface. (1) presents the current menu choices to the user. (2) displays incoming messages. There is an area for reading (3) and writing (4) e-mails. To allow quick selection and prevent confusion, the user chooses either the reply, compose, or next e-mail icon. The selection process is the same as for links on a web page. Addresses can be chosen from an address book created by the supervisor. Considering the fact that BCI users will generally read and write short messages, these two windows were placed next to each other, preventing the need to open new windows. E-mails are composed with a virtual keyboard.

Nessi includes an e-mail interface that allows the user to read and compose e-mails. A screenshot is shown in Figure 5.

To allow quick selection and prevent confusion, the user chooses either the reply, compose, or next e-mail icon. The selection process is the same as for links on a web page. Addresses can be chosen from an address book created by the supervisor. Considering the fact that BCI users will generally read and write short messages, these two windows were placed next to each other, preventing the need to open new windows. E-mails are composed with a virtual keyboard.

## 4. USER INTERFACE SIMULATIONS

**Figure 6:**
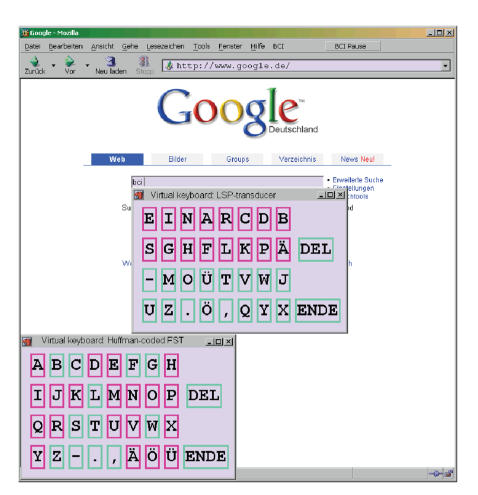
Virtual keyboards for entering text into web forms, displaying the LSP transducer (center) and the Huffman-coded transducer (bottom), which are used in the simulations.

**Figure 7:**
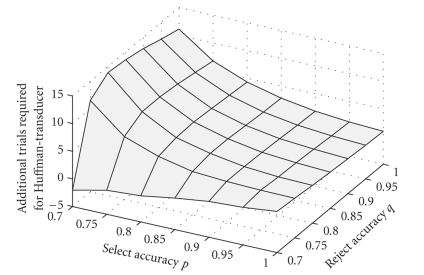
Number of additional brain responses to write a letter, required by the Huffman-transducer compared to the scanning transducer, as a function of the select and reject accuracies *p* and *q* (based on German letter frequencies).

Simulations were carried out to determine the interface's efficiency, given the user's accuracy of correct *selection* and correct *rejection* . As an example, the average number of brain responses needed to select a link on the page shown in Figure 1 is 16, if *p* = *q* = 0.75 . For spelling, the standard LSP transducer and a Huffman-coded transducer with *back* nodes on every second level were compared using Nessi's simulation mode. The average number of brain responses required to select a letter with the virtual keyboard (German language model), as shown in Figure 6, was simulated. The number of additional brain responses required for the Huffman-coded FST is displayed in Figure 7. A typical SCP or SMR brain response takes about 5 seconds.

Especially for low values of , the LSP transducer is more efficient than the Huffman-coded FST with *back* nodes. The difference is negligible for very low values of *p* and *q* . A further simulation showed that the placement of letters commonly found in LSP transducers, which is optimal for users with an accuracy of 100%, is suboptimal once *p* and *q* are below 1. If *p* and *q* are known for a particular user, an improved LSP transducer can be generated by the simulation module.

## 5. CONCLUSION AND DISCUSSION

Karim [10] have previously shown that an EEG-controlled web browser can help locked-in patients to regain a certain level of autonomy in the interaction with the outside world and thereby enhance their quality of life. However, this web browser required the user to select links from an alphabetical list, causing problems if the link names were identical or if they were unknown to the user (as in graphical links). These shortcomings have been resolved with the web browser Nessi. Graphical in-place markers are used instead of link text, whereby different brain responses correspond to frame colors placed around selectable items, enabling the user to select any link on a web page. Other interactive elements, such as e-mail and virtual keyboards, are also accessible and open up a wide range of hypertext-based applications to the user.

Moreover, the user interface has been optimized for low-bandwidth input. Even though classifier optimization is crucial to brain-computer interfaces, patients can benefit additionally from intelligent user interfaces. A language model was used to construct FSTs with options for error correction for entering URLs or text in web forms. We incorporated knowledge of the user's web page revisitation patterns to allow quick selection of pages that are visited often. Intelligent decisions are taken wherever possible, for example, links pointing to a common URL are subsumed in a single transducer state, and web pages without links result in Nessi returning to the previous page after the reading pause. A game was implemented to accustom users to the new interface and to increase motivation to use the interface.

Different types of transducers were compared by simulating the user input. The LSP transducer should be used for the virtual keyboards as well as link selection. However, it can be improved for known values of and . Note that the simulations do not consider factors such as user preferences or difficulties when generating the same brain response often in succession.

Future work will involve testing Nessi with patients communicating via the SCP or SMR paradigm, and testing a link selection interface for patients who prefer the P300 paradigm. In this case, each link is assigned to a letter, which is selected from a standard P300 speller matrix. Finally, we would like to evaluate the benefits of Nessi's user interface for other low-bandwidth users such as cerebral palsy patients or binary switch users.
